# Strapdown Airborne Gravimetry Quality Assessment Method Based on Single Survey Line Data: A Study by SGA-WZ02 Gravimeter

**DOI:** 10.3390/s18020360

**Published:** 2018-01-26

**Authors:** Minghao Wang, Meiping Wu, Juliang Cao, Kaidong Zhang, Shaokun Cai, Ruihang Yu

**Affiliations:** College of Mechatronics and Automation, National University of Defense Technology, Changsha 410073, China; wang900304@nudt.edu.cn (M.W.); jlcao@nudt.edu.cn (J.C.); zhangkaidong@nudt.edu.cn (K.Z.); shaokuncai@nudt.edu.cn (S.C.); yuruihang@nudt.edu.cn (R.Y.)

**Keywords:** airborne gravimetry, strapdown inertial navigation system, quality assessment, single survey line

## Abstract

Quality assessment is an important part in the strapdown airborne gravimetry. Root mean square error (RMSE) evaluation method is a classical way to evaluate the gravimetry quality, but classical evaluation methods are preconditioned by extra flight or reference data. Thus, a method, which is able to largely conquer the premises of classical quality assessment methods and can be used in single survey line, has been developed in this paper. According to theoretical analysis, the method chooses the stability of two horizontal attitude angles, horizontal specific force and vertical specific force as the determinants of quality assessment method. The actual data, collected by SGA-WZ02 from 13 flights 21 lines in certain survey, was used to build the model and elaborate the method. To substantiate the performance of the quality assessment model, the model is applied in extra repeat line flights from two surveys. Compared with internal RMSE, standard deviation of assessment residuals are 0.23 mGal and 0.16 mGal in two surveys, which shows that the quality assessment method is reliable and stricter. The extra flights are not necessary by specially arranging the route of flights. The method, summarized from SGA-WZ02, is a feasible approach to assess gravimetry quality using single line data and is also suitable for other strapdown gravimeters.

## 1. Introduction

The gravity information is crucial in the determination of geoid and resource exploration. Compared with gravimetry carriers, airborne gravimetry not only can satisfy the requirements for accuracy and resolution in different application fields but also is a high-efficiency way to collect gravity information [1,2]. Currently according to different principles, there are two types of airborne gravimeters [3,4]. One is platform airborne gravimeter, and the other is strapdown airborne gravimeter which is based on Strapdown Inertial Navigation System (SINS). Both platform airborne gravimeter and strapdown airborne gravimetry need Global Positioning System (GPS) to provide precise acceleration information. The AIRGrav and GT-2A gravimeter are two mature platform airborne gravimeters. The accuracy of AIRGrav system is superior to 1 mGal/2–4 km (1 mGal ≈ 10^−5^ m/s^2^) even under the turbulent conditions. Additionally, the accuracy of GT-2A airborne gravimetry can reach to 0.6 mGal/3 km [5,6,7]. Former research showed that strapdown airborne gravimetry could obtain a considerable precision compared to the platform airborne gravimeters in the high-resolution domain, the representatives are SISG and SGA-WZ [8,9,10]. What’s more, high accuracy strapdown inertial navigation system could be used as strapdown gravimeter as well after applying proper thermal compensation [11]. At the same time, research shows that the strapdown gravimeter can also be used for vector gravimetry [12,13,14]. Strapdown gravimeter uses a math platform instead of the physical platform to track the navigation coordinate, so strapdown airborne gravimeter has the advantages of lower cost, smaller sized and simpler structure than the platform gravimeters. What’s more, airborne gravimeter can be used in ground vehicle vector gravimetry and marine gravimetry through improving the algorithm [15,16,17,18].

Every careful measurement in science is always given with the probable error, so the quality assessment is an important aspect of airborne gravimetry to obtain high quality gravity disturbance data, when apply strapdown gravimetry into an actual survey [19]. The classical quality assessment methods not only accurately assess the dynamic performance and measurement consistency of airborne gravimeter, but also can reflect the gravimeter’s actual working state [20]. However, the classical quality assessment method needs the extra flights or reference data, which will become inefficiency and increase the cost of airborne gravimetry. So, the classical quality assessment method tends to the gravimeter performance evaluation methods and can’t efficiently evaluate the quality of single survey line or flight when it is done. A quality assessment algorithm based on the classical remove-compute-restore procedure is developed in recently research, which estimate not only the accuracy but also the error covariance function from a set of crossovers [21]. Meanwhile, how the surveyors estimate the quality of single survey line or flight in airborne gravimetry is a significant and practical problem. Actually, the property of airborne gravimetry stabilize at certain level and should be decided by the gravimeter itself when a flight is finished, so the parameters from data processing could describe the performance of airborne gravimetry in large degree. Based on this, a quality assessment method for strapdown airborne gravimetry has been developed to efficiently estimate the strapdown airborne gravimetry quality using the single line data. The original purpose of this method was to evaluate the single line quality of SGA-WZ02 during a regional gravimetry task in the northwest of China. SGA-WZ02 is the strapdown airborne gravimeter developed by National University of Defense Technology, which is based on the rich design experience of SGA-WZ01 [9,14,17,22]. The quality assessment method is similar to the gravimeter calibration and the determinants of quality assessment method are from the strapdown airborne gravimeter data processing. Both theoretical improvement and application of the new method to real flight data are presented in this paper. It should be noted that although the model was summarized from SGA-WZ02 gravimeter, the quality assessment method is suitable for other strapdown gravimeters to evaluate the single line gravimetry quality if they apply this method. Both advantages and disadvantages of the new quality assessment method are discussed in this paper.

## 2. Classical Root Mean Square Error Evaluation Methods for Airborne Gravimetry

The classical quality assessment methods need additional flight or extra information. The most common and classical quality assessment methods of airborne gravimetry are internal root mean square error (RMSE) and external RMSE evaluation methods. Evaluating internal RMSE needs repeat line flight to acquire gravity disturbance data in the same line or grid flight to acquire gravity disturbance data in the same point. The former is called repeat line internal RMSE and the latter is called the cross point internal RMSE. Equations (1) and (2) described the principle of repeat line internal RMSE. The principle of cross point internal RMSE was expressed by Equation (3) [20].

(1) $$\varepsilon_{j}=\pm \sqrt{\frac{\sum_{i=1}^{n}\delta_{ij}^{2}}{n}},(j=1,2,\cdots ,m)$$

(2) $$\varepsilon =\pm \sqrt{\frac{\sum_{j=1}^{m}\sum_{i=1}^{n}\delta_{ij}^{2}}{m\times n}}$$

where $\varepsilon_{j}$ is the internal RMSE of repeat line j, $\delta_{ij}$ is the different value between the mean value at point i and the value in repeat line j at point i, n is the total data point number of common part of repeat lines, m is the total line number of repeat line flight, ε is the internal RMSE of total repeat line.

(3) $$\varepsilon =\pm \sqrt{\frac{\sum_{j=1}^{m}\sum_{i=1}^{n}w_{ij}^{2}}{N}}$$

where ε is the internal RMSE of grid flights, $w_{ij}$ is the different value between line i and line j at cross point, N is the total data point number of grid lines.

The internal RMSE needs extra flight to assess quality of gravimetry, which will increase the cost of airborne gravimetry. Though the external RMSE doesn’t need extra flight, reference data from upward continuation or other reliable gravimeters is also needed to directly testify the performance of gravimeter. Compared with the internal RMSE, the result of external RMSE has a higher confidence level. The Equation (4) describes the principle of external RMSE [10]. In airborne gravimetry, the reference gravity disturbance data from the earth upward continuation is against the purpose of survey and the requirement of another airborne gravimeter is inefficient.

(4) $$\varepsilon =\pm \sqrt{\frac{\sum_{i=1}^{N}\omega_{i}^{2}}{N}}$$

where ε is the external RMSE, $\omega_{i}$ is the different value between reference data and measurement data at point i, N is the total reference data point number.

The repeat line internal RMSE can be used as the principle to illustrate the performance of certain airborne gravimeter and the cross point internal RMSE can evaluate the quality of the large area airborne gravimetry task. Another method, the external RMSE, can be applied to compare the data from certain airborne gravimeter with reference data to testify the performance of gravimeter in a different way from the internal RMSE. However, when the reference data or extra flights are not available, all these methods cannot directly evaluate the quality of single survey line as well.

## 3. Principle of Strapdown Airborne Gravimetry and Quality Assessment Method

**Figure 1:**
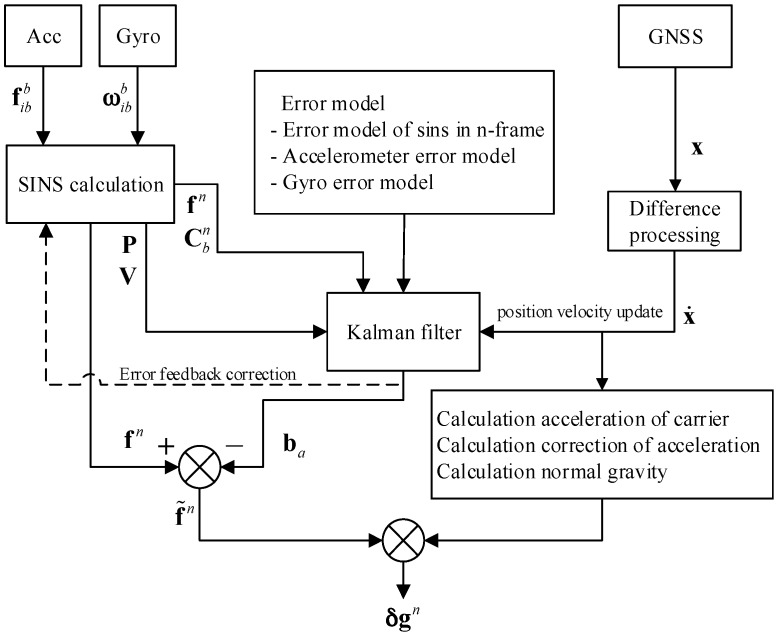
Data process flow chart of strapdown airborne gravimeter.

The purpose of gravimetry is to extract the mGal (≈10^−5^ m/s^2^) level gravity disturbance from the G (≈9.8 m/s^2^) level origin measurement data, which means the signal-to-noise ratio is very small. The purpose of gravimetry is to recover gravity disturbances with an accuracy of the order of 1 mGal ($1\;\mathrm{mGal}=10^{-5}\;m/s^{2}$) from a signal of the order of $10^{6}$ mGal, i.e. with an extremely small signal-to-noise ratio. The flow chart of strapdown airborne gravimeter data processing is shown in Figure 1 [9]. The strapdown airborne gravimeter can be regarded as a multiple inputs single output control system. The inputs of this control system are from inertial sensors and GPS, whereas the output is the gravity disturbance. As a control system, the strapdown airborne gravimeter also has a limited measurement ability and is related to the noise level of the system inputs. 

Furthermore, the principle for determining gravity anomaly in airborne gravimeter is shown in Equation (5) which is based on the Newton’s motion equation [9].

(5) $$\delta g^{n}={\dot{v}}_{e}^{n}+(\omega_{en}^{n}+2\omega_{ie}^{n})\times v_{e}^{n}-C_{b}^{n}f^{b}-\gamma^{n}$$

where ${\dot{v}}_{e}^{n}$ and $v_{e}^{n}$ are the acceleration and velocity of vehicle with respect to the earth, $f^{b}$ is the specific force measured by quartz flex accelerometers of a Strapdown Inertial Navigation System (SINS) in body frame (b-frame), $C_{b}^{n}$ is the transformation matrix which rotates from b-frame to n-frame, $\omega_{ie}^{n}$ is angular velocity of the earth respecting to the n-frame and $\omega_{en}^{n}$ is rotation rate of the ellipsoid n-frame due to the velocity of the vehicle relative to the ellipsoid, $\gamma^{n}$ is the normal gravity vector expressed in n-frame, $\delta g^{n}$ is the gravity disturbance vector expressed in n-frame.

In the Equation (5), the acceleration information of vehicle comes from GPS. The specific force and the transformation matrix rotating from b-frame to n-frame could be obtained from SINS. The accuracy of GPS was able to meet the requirement of airborne gravimetry in current condition [1,2,3], therefore the main error in strapdown airborne gravimeter comes from the inertial sensors. Considering the differential form of Equation (5) and only keeping the items related with SINS, the error model in three directions is shown in Equation (6) [23].

(6) $$d\delta g=C_{b}^{n}\delta f^{b}+\left[\psi \times \right]f^{n}=\left[\begin{array}{c} \delta f_{N}^{n}+\psi_{D}^{}f_{E}^{n}-\psi_{E}^{}f_{D}^{n}\\ \delta f_{E}^{n}-\psi_{D}^{}f_{N}^{n}-\psi_{N}^{}f_{D}^{n}\\ \delta f_{D}^{n}+\psi_{N}^{}f_{E}^{n}-\psi_{E}^{}f_{N}^{n} \end{array}\right]$$

where dδg is the gravity disturbance measurement error, $\delta f_{i},i=N,E,D$ are the specific force measurement errors in n-frame, $f_{i},i=N,E,D$ are the specific forces in n-frame and $\psi_{i},i=N,E,D$ are the attitude measurement errors in n-frame. 

Only considering the error in down direction, Equation (6) has shown that the accuracy of attitude measurement and specific force measurement are the key factors in strapdown airborne gravimetry. The error model of three-axis accelerometers unit in b-frame is shown in Equation (7), in which the error factors impacts the measurement through specific force inputs. In addition, gyro error factors influence the attitude measurement in a similar way with accelerometers error factors impacting the specific force measurement. So, larger input means larger measurement error in accelerometers when most of the error factors stabilize at certain levels, which is the same in the gyros. Furthermore, larger specific force errors from accelerometers and larger attitude rate errors from gyros will lead larger specific force errors and attitude errors in navigation-frame, which will eventually negative impact the airborne gravimetry result. In particular, airborne gravimetry mainly measures the change of gravity disturbance during the flight and is a relative measurement way, the bias of which is corrected by base point correction. Therefore, establishing the model between stability of airborne gravimeter inputs and gravimetry quality is a feasible and reliable way to estimate single line quality of airborne gravimeter. However, real inputs can’t be exactly obtained. Considering the error model shown in the third row of Equation (6), five factors, which were $\delta f_{D}$, $\psi_{N}$, $\psi_{E}$, $f_{E}$ and $f_{N}$, are all related with flight stability. First, larger $f_{D}$ leads to larger $\delta f_{D}$. Second, higher dynamic condition leads larger $\psi_{N}$ and $\psi_{E}$. Third, larger $f_{E}$ and $f_{N}$ amplify the error caused by $\psi_{N}$ and $\psi_{E}$. A case is that the airborne gravimeter performs better in the smooth airflow condition than in the turbulence airflow condition. Totally speaking, stability of roll angle, pitch angle, north specific force, east specific force and vertical specific force not only can describe the stability of flight but also have obvious relationship with gravimetry quality which can be regarded as the determinants in the estimation model.

(7) $$\left[\begin{array}{c} \Delta f_{x}\\ \Delta f_{y}\\ \Delta f_{z} \end{array}\right]=\left[\begin{array}{c} k_{0x}\\ k_{0y}\\ k_{0z} \end{array}\right]+\left[\begin{array}{ccc} k_{xx} & k_{xy} & k_{xz}\\ k_{yx} & k_{yy} & k_{yz}\\ k_{zx} & k_{zy} & k_{zz} \end{array}\right]\left[\begin{array}{c} f_{x}\\ f_{y}\\ f_{z} \end{array}\right]+\left[\begin{array}{c} n_{x}\\ n_{y}\\ n_{z} \end{array}\right]$$

where $\Delta f_{i},\left(i=x,y,z\right)$ is the measurement error of accelerometers, $k_{0i}\left(i=x,y,z\right)$ is the bias of the accelerometer, $k_{ii}\left(i=x,y,z\right)$ is the first-order scale factor error, $f_{i_{}}\left(i=x,y,z\right)$ is the specific force input of each accelerometer, $k_{ij}\left(i,j=x,y,z,i\neq j\right)$ is the cross-coupled error and $n_{i}\left(i=x,y,z\right)$ is the white noise.

To estimate the airborne gravimetry quality by the five determinants, the repeat line flight is also needed to establish the assessment model. However, the purpose of repeat line flight here is different from the common flight, which aimed at building the connection between airborne gravimetry quality and the determinants. The standard of quality assessment is established by repeat line flight, which is like the use of high precision three-axis turntable in SINS calibration. Once the estimation model between determinants and internal RMSE were established from repeat line flight, it can be used in other survey tasks to get estimated quality of airborne gravimetry. For the intuitive idea that the quality of airborne gravimetry should be decided by the gravimeter itself, a quality assessment method for strapdown airborne gravimetry is developed. The actual data used in method demonstration was collected during a regional gravimetry by SGA-WZ02 airborne gravimeter. The following part will introduce the survey conditions in detail.

## 4. Survey Description

**Figure 2:**
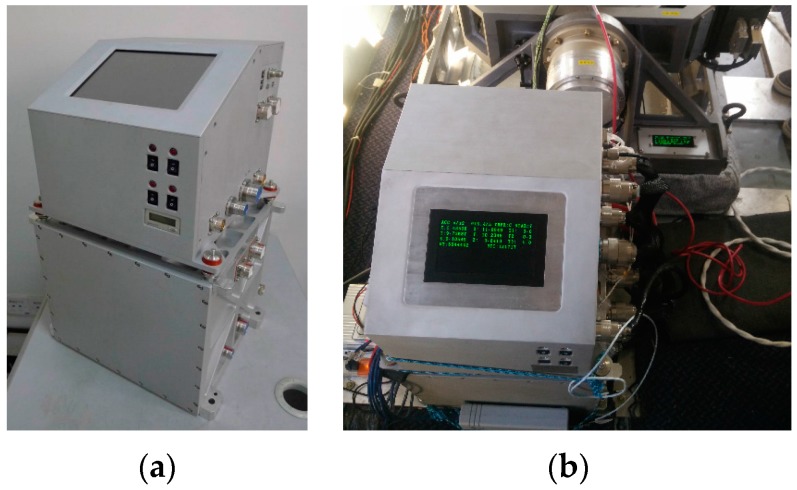
(**a**) The appearance of SGA-WZ02; (**b**) the SGA-WZ02 in the cabin.

The survey was carried out in the southwest of China's Xinjiang Province using SGA-WZ02 from September to October 2016. The properties of SGA-WZ02 were shown in Table 1. Figure 2 shows SGA-WZ02 airborne gravimeter. Three GPS receivers were used for the differential kinematic positioning, one of the receivers was located on the airplane and the other two were located on the roof as ground stations. All the GPS receivers installed on the ground and in the airplane were Trimble BD930. The GPS positioning accuracy was better than ±0.1 m, the accuracy of the velocity determination was better than 0.05 m/s, when applied differential kinematic positioning by Waypoint Software. Lever arm, which was used to transform the sensitive center of GPS to SINS sensitive center, was ${\left[1.90,-0.22,-1.60\right]}^{\prime }\;m$ in the INS body frame.

**Figure 3:**
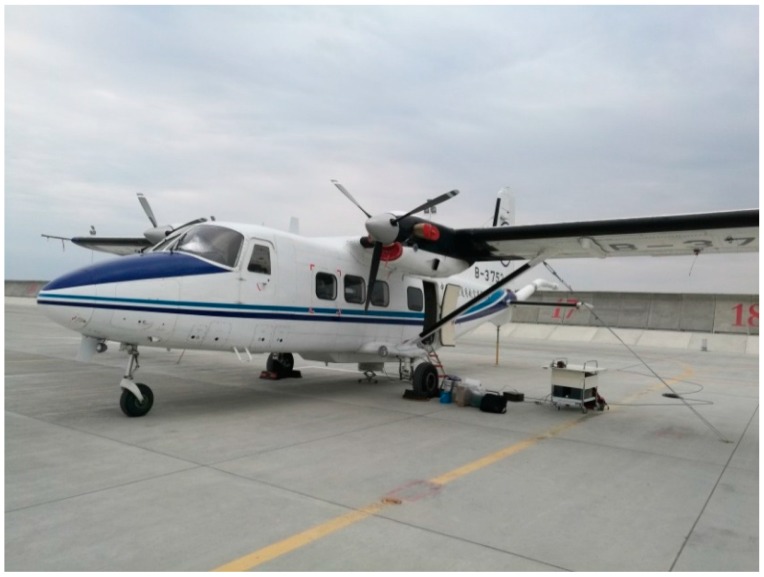
Appearance of aircraft Y-12.

**Figure 4:**
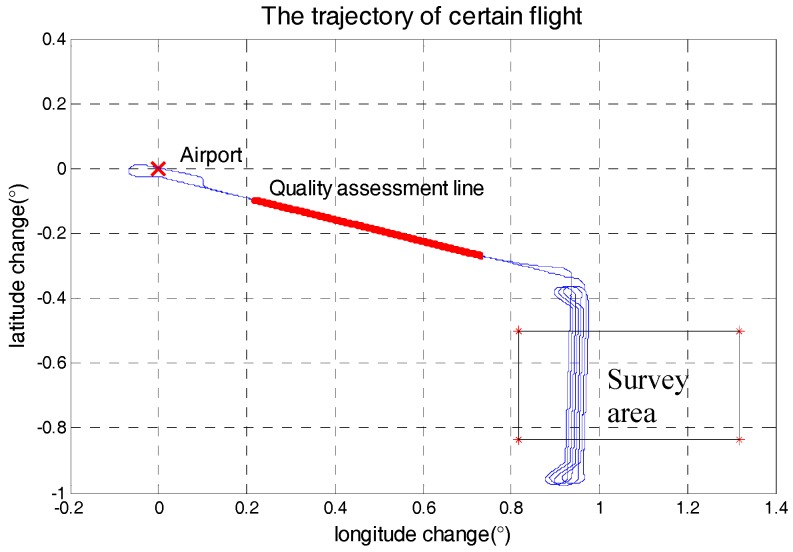
Path of certain flight.

The gravimetry system was mounted on a Y-12 aircraft, which was a fixed-wing aircraft without an autopilot. The detail characters of flight were shown in Table 2. The Y-12 is shown in Figure 3. The survey area was about 1600 km^2^, in which the longitude range was 0.5° and the latitude range was about 0.33°. Each survey line was about 40 km, whose ends were extended 5 km to eliminate the boundary effect of low-pass filter. In this regional airborne gravimetry task, the maximum distance between survey area and airport was less than 140 km. The flight path from airport to survey area was fixed to collect repeat line data, which could be called quality assessment line. Figure 4 is the path of certain flight and path of quality assessment line is red highlight.

**Figure 5:**
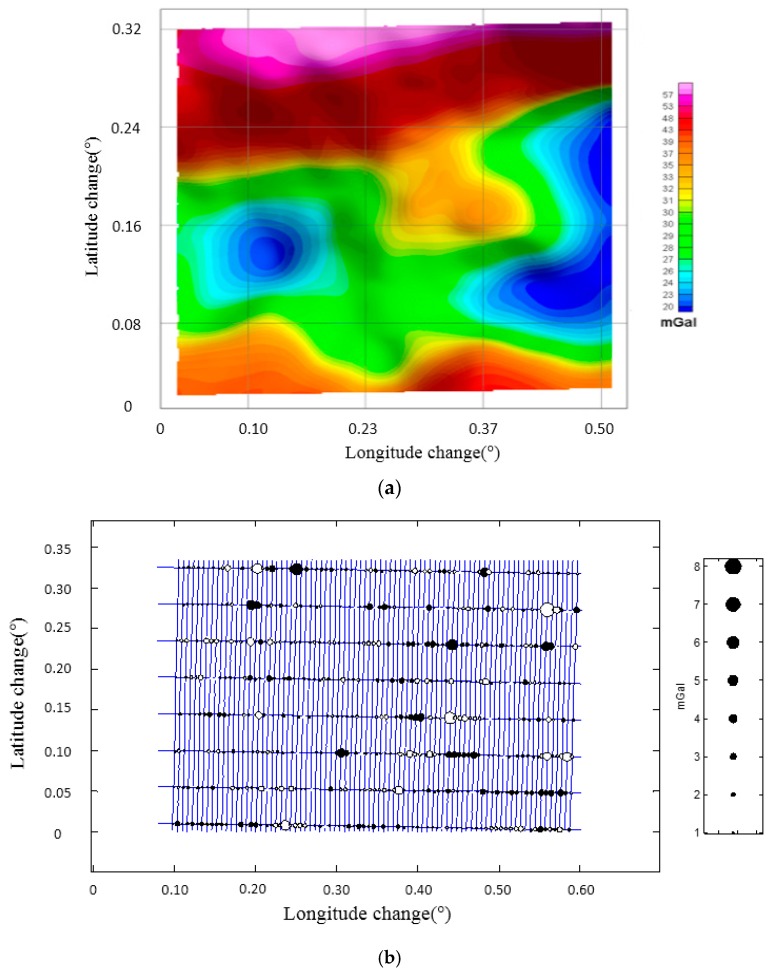
(**a**) Gravity disturbance in the survey area after adjustment; (**b**) Cross-point differences in the survey area after adjustment.

To cover the survey area, there were 82 north-south lines and 8 east-west cross lines. The interval of north-south lines was 500 m and the interval of east-west lines was 5000 m. In addition, there were also several experimental flights, for example undulated flights, during the survey. So, this survey totally contained 21 separate flights. Based on the cross-point internal quality assessment, the total accuracy of this survey was 2.09 mGal in the resolution of 4.8 km. After cross-point adjustment, the accuracy could reach 1.30 mGal. Figure 5 shows the gravity disturbance and cross-point differences in the survey area after adjustment. 

The use of cross point internal RMSE could evaluate the truly accuracy of system and the total survey quality. However, as mentioned, cross point internal RMSE needs extra flight and more extra flights mean more comprehensive evaluation. When the cross points are not available, how to assess the single line quality should be considered, which means the quality assessment method is quite needed. Let assume that such a situation, the survey lines were added outside the arranged area where cross-over points were unavailable. Under above conditions, the single line quality assessment method is the proper way to assess the new lines’ quality. In addition, single line quality assessment method is also helpful in the airborne gravimetry quality control. In the next section, the relationships between quality assessment determinants and internal RMSE in this regional airborne gravimetry task were given by linear fitting. The single line quality assessment model for SGA-WZ02 was built by weighted linear fitting results. What needs to be noted is that the model was specially established for SGA-WZ02 while the method could be also generalized to other strapdown gravimeter. 

## 5. Quality Assessment Model Development

**Figure 6:**
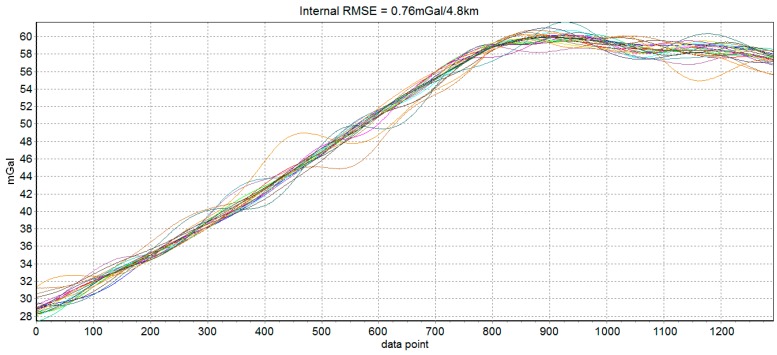
Measurement result of 21 quality assessment lines.

The error in strapdown airborne scalar gravimetry was mainly originated from the specific force errors in three directions and two horizontal attitudes errors. To analyze the relationship between the stability of five determinants and gravimetry quality, standard deviation of five determinants and gravity disturbance internal RMSE in quality assessment lines should be calculated. For the reason of flight path conflict, several flights did not finish the original plan, so the data from 21 quality assessment lines in 13 flights was collected and applied in the model establishment which was shown in Table 3. From Table 3, we can see that larger standard deviations of five determinants usually mean worse gravimetry quality, such as line 10-1 and 10-2, and small standard deviations usually mean better gravimetry quality, such as line 1-2 5-1 and 13-1. The gravimetry results of 21 quality assessment are shown in Figure 6. Most of quality assessment lines show good results and internal RMSE of these lines is 0.76 mGal under the resolution of 4.8 km. 

**Figure 7:**
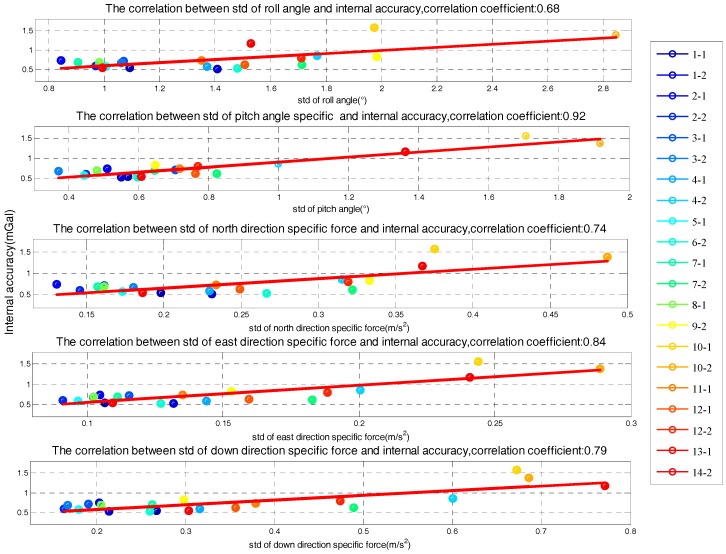
Correlation between determinants and internal root mean square error (RMSE).

**Figure 8:**
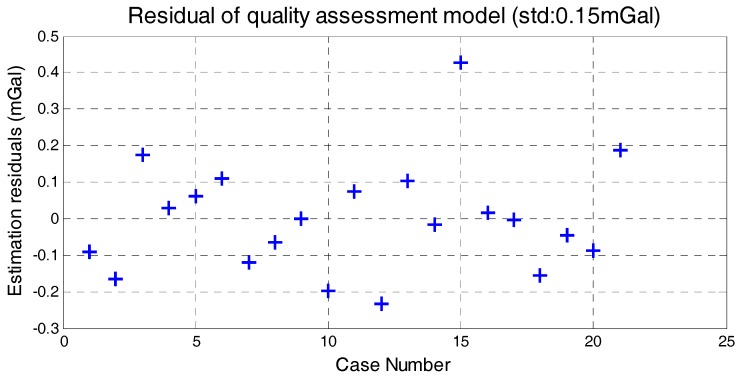
Model residuals.

The correlation between the stability of five factors and internal RMSE is shown in Figure 7, where *x*-axis is the standard deviation of determinant and *y*-axis is internal RMSE. The red line in each subfigure is obtained by linear fitting and the correlation coefficients are also given. Correlations between the determinants’ standard deviation and internal RMSE are all significant. Thus, it is reasonable to estimate the quality of survey line through the standard of five determinants. Based on linear fitting results in Figure 7, relationship model between the five factors and internal RMSE is built by weighted method, which is shown in Equation (8). The model residuals are shown in Figure 8. We can see that quality assessment model has shown good performance and the maximum residual is 0.43 mGal in case 15.

(8) $$\begin{array}{cc} \varepsilon & =\sum_{\begin{array}{l} x=\varphi_{n},\varphi_{e},\\ f_{n},f_{e},f_{d} \end{array}}\frac{1/R_{x}}{R_{0}}(p_{1}^{x}\mathrm{std}(x)+p_{0}^{x})\;(R_{0}=\sum_{\begin{array}{l} x=\varphi_{n},\varphi_{e},\\ f_{n},f_{e},f_{d} \end{array}}\frac{1}{R_{x}})\\ & =0.247+0.192\mathrm{std}(\varphi_{n})+0.235\mathrm{std}(\varphi_{e})+\\ & 0.057\mathrm{std}(f_{n})+0.025\mathrm{std}(f_{e})+0.129\mathrm{std}(f_{d}) \end{array}$$

where ε is the quality estimation result of single survey line, $\varphi_{n}$ is the roll angle, $\varphi_{e}$ is the pitch angle, $f_{n}$ is the north direction specific force, $f_{e}$ is the east direction specific force, $f_{d}$ is the down direction specific force, $R_{x},x=\varphi_{n},\varphi_{e},f_{n},f_{e},f_{d}$ are the variances of linear fitting results, $p_{1}^{x},x=\varphi_{n},\varphi_{e},f_{n},f_{e},f_{d}$ are the first order linear fitting coefficients and $p_{0}^{x},x=\varphi_{n},\varphi_{e},f_{n},f_{e},f_{d}$ are the zero order linear fitting coefficients.

**Figure 9:**
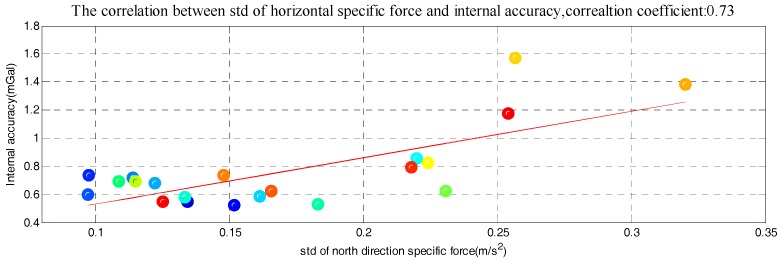
The relation between horizontal specific force and internal RMSE.

**Figure 10:**
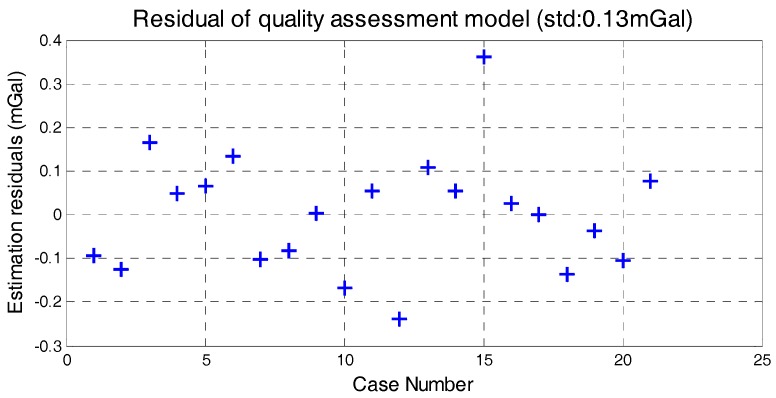
Residuals of new model.

However, the universality and applicability of model may be influenced by flight direction. It was because the stabilities of two horizontal specific force were related to the flight direction. So the north specific force and east specific force can be combined as one horizontal specific force. Equation (9) has shown how we got horizontal specific force. Relationship between the horizontal specific force standard deviation and internal RMSE is shown in Figure 9. The correlation between horizontal specific force standard deviation and internal RMSE is also significant. Based on new linear fitting results, the estimation model is rebuilt, which is shown in Equation (10). The residuals of new model are shown in Figure 10. Combining the north specific force with east specific force, the applicability of this model is promoted and also has good performance.

(9) $$f_{h}=\sqrt{f_{n}^{2}+f_{e}^{2}}$$

where $f_{h}$ is the down direction specific force, $f_{n}$ is the north direction specific force and $f_{e}$ is the east direction specific force.

(10) $$\begin{array}{cc} \varepsilon & =\sum_{\begin{array}{l} x=\varphi_{n},\varphi_{e},\\ f_{h},f_{d} \end{array}}\frac{1/R_{x}}{R_{0}}(p_{1}^{x}\mathrm{std}(x)+p_{0}^{x})\;(R_{0}=\sum_{\begin{array}{l} x=\varphi_{n},\varphi_{e},\\ f_{h},f_{d} \end{array}}\frac{1}{R_{x}})\\ & =0.266+0.055\mathrm{std}(\varphi_{n})+0.310\mathrm{std}(\varphi_{e})+\\ & 0.5441\mathrm{std}(f_{h})+0.244\mathrm{std}(f_{d}) \end{array}$$

where ε is quality estimation result of survey line, $\varphi_{n}$ is the roll angle, $\varphi_{e}$ is the pitch angle, $f_{h}$ is the horizontal specific force and $f_{d}$ is the down direction specific force $R_{x},x=\varphi_{n},\varphi_{e},f_{h},f_{d}$ are variances of linear fitting results, $p_{1}^{x},x=\varphi_{n},\varphi_{e},f_{h},f_{d}$ are the first order linear fitting coefficients and $p_{0}^{x},x=\varphi_{n},\varphi_{e},f_{h},f_{d}$ are the zero order linear fitting coefficients.

Based on the strong correlation, the assessment model in SGA-WZ02 is obtained by weighted linear fitting. After fusion the north and east specific force, the effect of flight direction is eliminated to a large extent in quality assessment. The model is specially established for SGA-WZ02 gravimeter assessing the gravimetry quality, but a similar model can also be obtained by other strapdown gravimeter during a gravimetry task if the same method is used. In the next section, data from other repeat lines will be used to testify the quality assessment model performance. 

## 6. Quality Assessment Model Test

**Figure 11:**
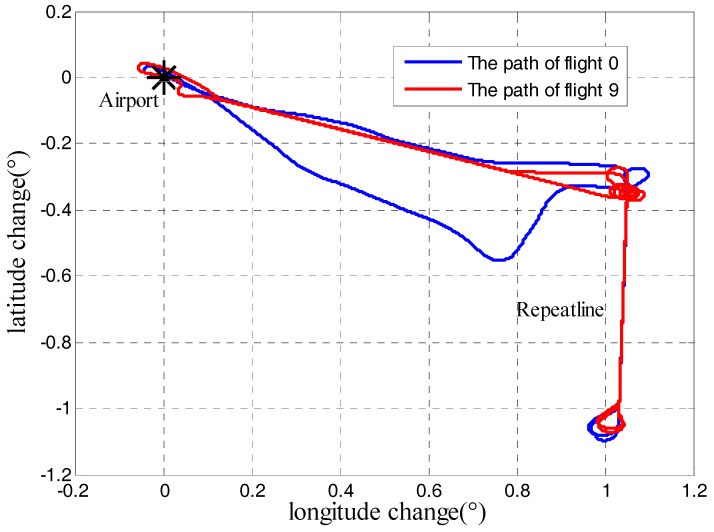
Paths of two repeat line flight.

**Figure 12:**
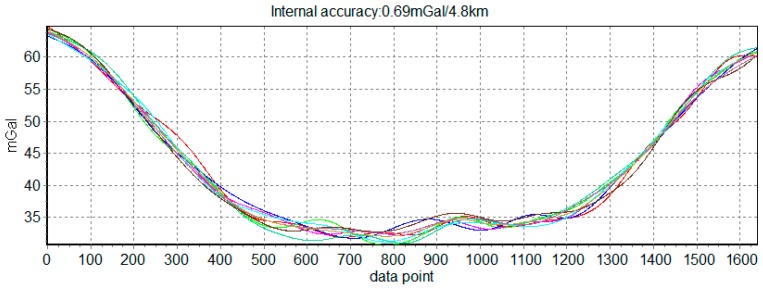
Measurement result of 8 repeat lines.

To test applicability and veracity of quality assessment model, repeat flights were used, whose flight paths are different from the highlight path shown in Figure 4. The first test flight is a repeat line flight before the survey flight 1, which is to initially inspect the gravimeter’s working performance and can be numbered flight 0. The second test flight is flight 9. There are a total of eight repeat lines and two different flight paths are shown in Figure 11. The measurement result of 8 repeat lines was shown in Figure 12. The internal RMSE is 0.69 mGal/4.8 km, so SGA-WZ02 gravimeter still shows high accuracy.

**Figure 13:**
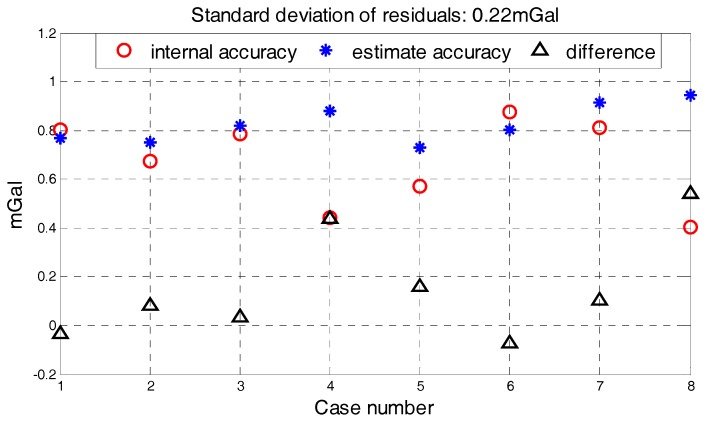
Differences between the quality assessment results and internal RMSE.

The standard deviation of four determinants and internal RMSE were shown in Table 4. Compared with the data in Table 3, four determinants in Table 4 represent the good flight stability. Using the quality assessment model shown in Equation (10) and the data from Table 4, quality assessment results of 8 lines in two flights are shown in Table 5 and differences between the quality assessment results and internal RMSE is shown in Figure 13. The standard deviation of residuals is 0.22 mGal and the largest estimation error is about 0.54 mGal in Figure 13. The good performance of quality assessment model can be seen from Table 5 and Figure 13. At the same time, the quality assessment results are larger than the internal RMSE in most cases, so we can conclude that the quality assessment result obtained by model is a conservative indicator for quality assessment. The quality requirement of this gravimetry task is superior to 1 mGal under 4.8 km resolution and the survey lines which do not meet the accuracy requirement need to be remeasured. Both quality assessment results and internal RMSE in Table 5 satisfy the accuracy requirement, which confirm the performance of quality assessment model. So, if single survey line’s quality assessment result is less than 1, it represents the result of this line satisfies the quality requirement and remeasurement does not need in a large degree. The quality assessment result obtained by the model is a complement for the classical methods during the gravimetry task.

**Figure 14:**
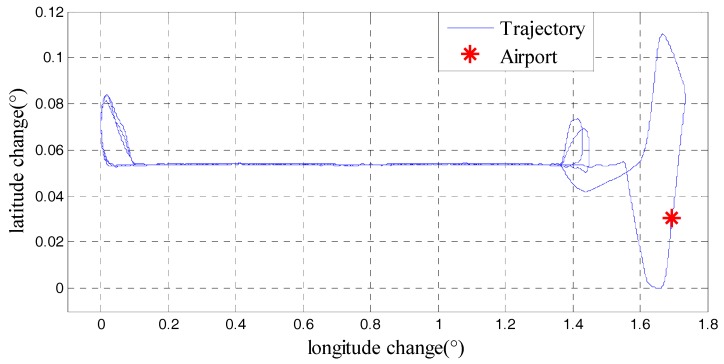
Trajectory of Neimeng flight.

**Figure 15:**
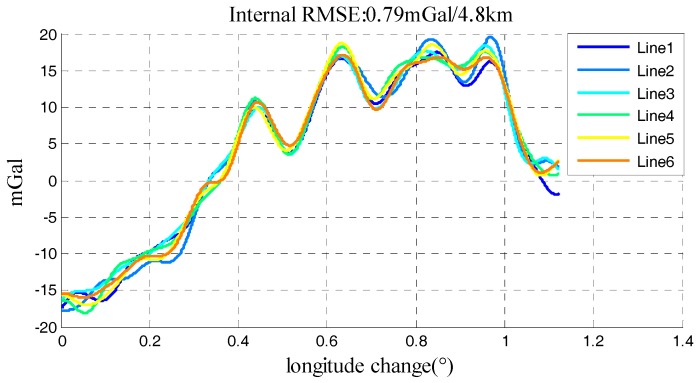
Gravity disturbance results in Neimeng flight.

Furthermore, a repeat line flight in another gravimetry survey was applied to further test the performance of model and the generality of the method. This repeat line flight which carried SGA-WZ02 gravimeter was implemented in Neimeng province of china in June 2015. Although there were several calibrations on SGA-WZ02 between the two surveys, the core sensors were still the same. Flight direction in this survey was east to west and the aircraft was also Y-12. Table 6 showed the characters of Neimeng flight and trajectory was shown in Figure 14. And the final gravity disturbance results in Neimeng flight were shown in Figure 15 which was 0.79 mGal under resolution of 4.8 km.

**Figure 16:**
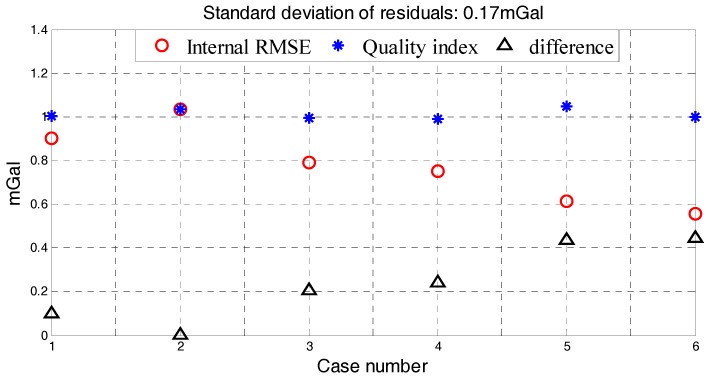
Different between internal RMSE and Quality assessment results in Neimeng flight.

The standard deviation of four factors, internal RMSE and quality assessment results in Neimeng flight were shown in Table 7. Quality assessment results of 6 lines in Neimeng flight were shown in Figure 16. The standard deviation of residuals was 0.17 mGal and the largest estimation error was 0.45 mGal in Figure 16. The quality assessment result is still an exacting index for quality assessment. The Neimeng flight’s quality assessment results show the performance of established model when applying in different survey. Compared four determinants in Table 4 and Table 7, the stability of flight in Neimeng is worse than that in Xinjiang. As a result, the performance of SGA-WZ02 in Xinjiang is better than that in Neimeng, which could be seen from the quality assessment results and internal RMSE. 

In conclusion, quality assessment model built the connection between the single survey line data in strapdown gravimeter and the quality of gravimetry. Compared with internal RMSE, quality assessment model was conservative and strict in these two surveys. The quality assessment results in different surveys indicated that quality assessment model could be used to estimate the performance of strapdown airborne gravimeter and could be regarded as an important reference index for surveyors to control the gravimetry process as well. As a case, the shown method also could be applied to other strapdown gravimeters.

## 7. Discussion and Conclusions

This research developed an efficient and pervasive method for strapdown airborne gravimetry quality assessment using single survey line data. The single survey line data used in the method were roll angle, pitch angle, integrated horizontal specific force, and vertical specific force, which were regarded as quality assessment determinants and could also quantify the flight stability during the survey task. Taking advantage of two different gravimetry surveys, the method applicability and generality have been testified and both got promising results. Once the strapdown gravimeter quality assessment model established, it could not only be used to assess the quality of gravimetry in this survey but also in other surveys with similar conditions. 

The quality assessment method can be described by four steps.

Step 1: Collect enough repeat line data which can be obtained from different flights.
Step 2: Calculate the standard deviation of four determinants and obtain the internal RMSE results in those repeat lines.
Step 3: Establish the estimation model whose inputs are the standard deviation of four determinants and output is quality estimation result.
Step 4: Extract standard deviation of four determinants in new survey line and apply the estimation model obtained in step 3 to assess the quality of survey line.

Taking advantage of the above four steps, this method developed by SGA-WZ02 could also be applied for other strapdown gravimeters.

Applied to real data in different surveys, the practicality of the quality assessment method has been validated. The merits of this quality assessment method are as follows. First, using the model is a convenient and low-cost way to obtain a reliable quality assessment result for gravimetry. Second, besides examining the performance and work state of gravimeter, repeat line flight also can be used to build quality assessment model, which expands the function of repeat line flight. Third, this new assessment method could complement the traditional classical methods and help ensure the quality of the measurements during airborne gravimetry and also allowed a more complete assessment of the entire gravity measurement. Fourth, once the model was established, it could be applied and improved in another different survey task. Fifth, the new assessment method has shown the advantage of strapdown airborne gravimeter in data mining. In addition, extra repeat flights plans can be avoided by trajectory planning during establishing a quality assessment model, and corrective models can be constantly repaired during the survey mission. However, disadvantage of new method was that this method just offered a conservatively estimated value of survey quality. In the future research, the credibility of estimation quality should also receive more attention.

## Figures and Tables

**Table 1:** Properties of SGA-WZ02.

Properties	Details
Static precision	0.5 mGal (24 h)
Max overload	4 g
Working temperature	−10–50 ℃
Weight	45 kg
Dimension	460 mm × 350 mm × 580 mm
Stable power consumption	<150 w

**Table 2:** The characters of flight.

Character	Details
Aircraft type	Y-12
Topography	Gentle hilly terrain
Atmosphere conditions	Variety
Altitude above standard ellipsoid	1600 m
Flight speed	60 m/s
Trace control method	Line flight control
Sampling frequency of GPS	2 Hz
Sampling frequency of SINS	200 Hz

**Table 3:** Standard deviation of five factors and internal RMSE in 21 lines.

Line Number	Roll Angle (°)	Pitch Angle (°)	$f_{n}$ ($m/s^{2}$)	$f_{e}$ ($m/s^{2}$)	$f_{d}$ ($m/s^{2}$)	RMSE (mGal)
1-1	1.092	0.568	0.198	0.106	0.267	0.547
1-2	1.408	0.549	0.231	0.132	0.214	0.524
2-1	0.844	0.510	0.130	0.105	0.203	0.739
2-2	0.968	0.449	0.145	0.091	0.163	0.599
3-1	1.070	0.704	0.161	0.115	0.191	0.717
3-2	1.061	0.371	0.180	0.102	0.167	0.681
4-1	1.371	0.596	0.230	0.144	0.316	0.588
4-2	1.767	0.997	0.315	0.200	0.601	0.856
5-1	1.006	0.443	0.173	0.097	0.179	0.579
6-2	1.480	0.597	0.267	0.127	0.260	0.527
7-1	0.905	0.645	0.157	0.111	0.262	0.693
7-2	1.713	0.822	0.322	0.183	0.489	0.621
8-1	0.981	0.479	0.161	0.102	0.205	0.689
9-2	1.983	0.646	0.333	0.153	0.298	0.826
10-1	1.974	1.705	0.375	0.244	0.672	1.570
10-2	2.846	1.917	0.487	0.289	0.686	1.384
11-1	1.351	0.716	0.234	0.135	0.378	0.737
12-1	1.507	0.762	0.249	0.160	0.356	0.625
12-2	1.710	0.768	0.319	0.188	0.474	0.795
13-1	0.992	0.607	0.186	0.109	0.303	0.545
13-2	1.529	1.361	0.367	0.241	0.771	1.175

**Table 4:** Standard deviation of four factors and internal RMSE in two repeat line flight.

Line Number	Roll Angle (°)	Pitch Angle (°)	$f_{h}$ ($m/s^{2}$)	$f_{d}$ ($m/s^{2}$)	RMSE (mGal)
0-1	1.188	0.876	0.132	0.378	0.803
0-2	0.897	0.864	0.121	0.425	0.674
0-3	1.388	0.933	0.162	0.402	0.786
0-4	1.143	1.026	0.183	0.549	0.444
9-1	1.345	0.681	0.145	0.398	0.571
9-2	1.616	0.815	0.177	0.406	0.875
9-3	1.458	0.942	0.225	0.622	0.810
9-4	1.989	0.966	0.235	0.581	0.405

**Table 5:** Standard deviation of four factors and internal RMSE in two repeat line flight.

Line Number	0-1	0-2	0-3	0-4	9-1	9-2	9-3	9-4
Quality assessment results	0.767	0.753	0.818	0.881	0.727	0.804	0.913	0.945
Internal RMSE	0.803	0.674	0.786	0.444	0.571	0.875	0.810	0.405

(Unit: mGal).

**Table 6:** Characters of Neimeng flight.

Character	Details
Aircraft type	Y-12
Topography	Plain
Atmosphere conditions	Turbulence
Altitude above standard ellipsoid	1550 m
Flight speed	60 m/s
Trace control method	Line flight control
Sampling frequency of GPS	2 Hz
Sampling frequency of SINS	200 Hz

**Table 7:** The standard deviation of four factors and internal RMSE in Neimeng flight.

Line Number	Roll Angle (°)	Pitch Angle (°)	$f_{h}$ ($m/s^{2}$)	$f_{d}$ ($m/s^{2}$)	RMSE (mGal)	Quality Assessment Result
1	1.562	1.142	0.231	0.703	0.903	1.00
2	1.057	1.435	0.196	0.652	1.034	1.04
3	1.336	1.168	0.227	0.696	0.792	0.99
4	1.075	1.400	0.173	0.560	0.751	0.99
5	1.384	1.353	0.252	0.614	0.613	1.05
6	0.873	1.530	0.180	0.452	0.554	0.99

## References

[1] Jekeli C. (1994). Airborne vector gravimetry using precise, position-aided inertial measurement units. J. Geod..

[2] Schwarz K.P., Wei M., Sünkel H., Marson I. (1995). Some unsolved problems in airborne gravimetry. Gravity and Geoid.

[3] Glennie C., Schwarz K.P. (2000). A comparison of stable platform and strapdown airborne gravity. J. Geod..

[4] Bruton A.M., Hammada Y., Ferguson S., Schwarz K.P., Wei M., Halpenny J. A comparison of inertial platform, damped 2-axis platform and strapdown airborne gravimetry. Proceedings of the International Symposium on Kinematic Systems in Geodesy, Geomatics and Navigation.

[5] Sander S., Argyle M., Elieff S., Ferguson S., Lavoie V., Sander L. The AIRGrav airborne gravity system. Proceedings of the ASEG-PESA Airborne Gravity Workshop.

[6] Berzhitsky V.N., Bolotin Y.V., Golovan A.A., Ilyin V.N., Parusnikov N.A., Smoller Y.L., Yurist S.S. (2002). GT-1A Inertial Gravimeter System. Results of Flight Tests.

[7] Olson D. GT-1A and GT-2A airborne gravimeters: Improvements in design, operation, and processing from 2003 to 2010. Proceedings of the ASEG-PESA Airborne Gravity 2010 Workshop.

[8] Wei M., Schwarz K.P. (1998). Flight test results from a strapdown airborne gravity system. J. Geod..

[9] Cai S.K., Wu M.P., Zhang K.D., Cao J.L., Tuo Z.H., Huang Y.M. (2013). The first airborne scalar gravimetry system based on SINS/DGPS in China. Sci. Chin: Earth Sci..

[10] Huang Y., Olesen A.V., Wu M., Zhang K. (2012). SGA-WZ: A New Strapdown Airborne Gravimeter. Sensors.

[11] Becker D., Nielsen J.E., Ayres-Sampaio D., Forsberg R., Becker M., Bastos L. (2015). Drift reduction in strapdown airborne gravimetry using a simple thermal correction. J. Geod..

[12] Kwon J.H., Jekeli C. (2001). A new approach for airborne vector gravimetry using GPS/INS. J. Geod..

[13] Senobari M.S. (2010). New results in airborne vector gravimetry using strapdown INS/DGPS. J. Geod..

[14] Cai S.K., Zhang K., Wu M. (2013). Improving airborne strapdown vector gravimetry using stabilized horizontal components. J. Appl. Geophys..

[15] Li X., Jekeli C. Ground-vehicle INS/GPS vector gravimetry assessment using repeated traverses in montana. Proceedings of the 1st International Symposium of the International Gravity Field Service.

[16] Li X. (2007). Moving Base INS/GPS Vector Gravimetry on a Land Vehicle. Ph.D. Thesis.

[17] Yu R., Wu M., Zhang K., Cai S., Cao J., Wang M., Wang L. (2017). A New Method for Land Vehicle Gravimetry Using SINS/VEL. Sensors.

[18] Cai S.K., Tie J.B., Zhang K.D., Cao J.L., Wu M.P. (2017). Marine gravimetry using the strapdown gravimeter SGA-WZ. Mar. Geophys. Res..

[19] Li X. (2011). Strapdown INS/DGPS airborne gravimetry tests in the Gulf of Mexico. J. Geod..

[20] Guo Z.H., Xiong S.Q., Zhou J.X., Zhou X.H. (2008). The research on quality evaluation method of test repeat lines in airborne gravity survey. Chin. J. Geophys..

[21] Sampietro D., Capponi M., Mansi A.H., Gatti A., Marchetti P., Sansò F. (2017). Space-Wise approach for airborne gravity data modelling. J. Geod..

[22] Cao J., Wang M., Cai S., Zhang K., Cong D., Wu M. (2015). Optimized Design of the SGA-WZ Strapdown Airborne Gravimeter Temperature Control System. Sensors.

[23] Cai S.K. (2014). The Research about Airborne Vector Gravimeter and Methods of Errors Separation. Ph.D. Thesis.

